# Constructing and implementing a performance evaluation indicator set for artificial intelligence decision support systems in pediatric outpatient clinics: an observational study

**DOI:** 10.1038/s41598-024-64893-w

**Published:** 2024-06-24

**Authors:** Yingwen Wang, Weijia Fu, Yuejie Zhang, Daoyang Wang, Ying Gu, Weibing Wang, Hong Xu, Xiaoling Ge, Chengjie Ye, Jinwu Fang, Ling Su, Jiayu Wang, Wen He, Xiaobo Zhang, Rui Feng

**Affiliations:** 1 Nursing Department, Children’s Hospital of Fudan University, Shanghai, 201102 China; 2 Medical Information Center, Children’s Hospital of Fudan University, Shanghai, 201102 China; 3 School of Computer Science, Fudan University, Shanghai, 200438 China; 4 School of Public, Health Fudan University, Shanghai, 200032 China; 5 Nephrology Department, Children’s Hospital of Fudan University, Shanghai, 201102 China; 6 Statistical and Data Management Center, Children’s Hospital of Fudan University, Shanghai, 201102 China; 7 National Health Commission Key Laboratory of Neonatal Diseases (Fudan University), Children’s Hospital of Fudan University, Shanghai, 201102 China; 8 Respiratory Department, Children’s Hospital of Fudan University, Shanghai, 201102 China; 9 School of Computer Science, Fudan University, 2005 Songhu Road, Shanghai, 200438 China

**Keywords:** Health care, Engineering

## Abstract

Artificial intelligence (AI) decision support systems in pediatric healthcare have a complex application background. As an AI decision support system (AI-DSS) can be costly, once applied, it is crucial to focus on its performance, interpret its success, and then monitor and update it to ensure ongoing success consistently. Therefore, a set of evaluation indicators was explicitly developed for AI-DSS in pediatric healthcare, enabling continuous and systematic performance monitoring. The study unfolded in two stages. The first stage encompassed establishing the evaluation indicator set through a literature review, a focus group interview, and expert consultation using the Delphi method. In the second stage, weight analysis was conducted. Subjective weights were calculated based on expert opinions through analytic hierarchy process, while objective weights were determined using the entropy weight method. Subsequently, subject and object weights were synthesized to form the combined weight. In the two rounds of expert consultation, the authority coefficients were 0.834 and 0.846, Kendall's coordination coefficient was 0.135 in Round 1 and 0.312 in Round 2. The final evaluation indicator set has three first-class indicators, fifteen second-class indicators, and forty-seven third-class indicators. Indicator I-1(Organizational performance) carries the highest weight, followed by Indicator I-2(Societal performance) and Indicator I-3(User experience performance) in the objective and combined weights. Conversely, 'Societal performance' holds the most weight among the subjective weights, followed by 'Organizational performance' and 'User experience performance'. In this study, a comprehensive and specialized set of evaluation indicators for the AI-DSS in the pediatric outpatient clinic was established, and then implemented. Continuous evaluation still requires long-term data collection to optimize the weight proportions of the established indicators.

## Introduction

In April of 2018, the US Food and Drug Administration approved the first Artificial intelligence (AI) device to detect eye disease in adults patients with diabetes^1^. The emergence of AI-based technology has garnered significant attention across various domains, particularly in advancing clinical support system, thereby reshaping medical care^2^. These advancements encompass a spectrum of AI applications, ranging from preventive strategies development^3,4^, image-based diagnosis^5–7^, nursing and therapy workflows improvement^8,9^. In China, where pediatric outpatient clinics grapple with perpetual overcrowding, AI-DSS has emerged as a valuable tool, particularly evident during the COVID-19 epidemic^10^, aiding in expediting disease diagnosis and treatment, and mitigating patient wait times^11,12^. However, the predominant focus on technical prowess during AI development often sidelines considerations for seamless integration into real-world workflows and the practical value of these innovations. Such oversights may only surface during clinical evaluations, as training datasets are meticulously curated to eliminate imperfect samples^13^. Moreover, AI-DSS in clinical settings can pose both costly and occasionally disruptive, highlighting the imperative to dynamically evaluate their efficacy post-adoption.

The rigorous evaluation of information system's quality and impact were initially proposed in the 1980s, encompassing every stage of its lifecycle, from design and development to selection and utilization. Initially, such publications focused on revealing techniques for assessing effectiveness in controlled laboratory settings. Subsequent papers delved into real-world testing in clinical environments, analyzing their impact on the structure, process, and outcome of healthcare delivery^14–18^. With the proliferation of AI-DSS in clinical practice, subsequent papers aimed at assessing the clinical impacts of them^8,19–22^.

Currently, various frameworks and guidelines exist for grouping indicators to evaluate AI system performance. The Delone and Mclean(D&M) Information System (IS) Success Model was well-known as a framework and model for measuring the variables in IS research in healthcare settings^23^. It was proposed to conceptualize and operationalize IS success^24^. Modified and extended, it now provides components of system quality, information quality, service quality, intention to use, user satisfaction, and net benefits, all interrelated^25–29^. Meanwhile, they can also be characterized as technology, task, user, organization, and environment, mainly focusing on the appraised objects^30,31^. The Guideline for Good Evaluation Practice in Health Informatics (GEP-HI) was developed to plan and conduct scientifically robust evaluation studies in healthcare^32^. Additionally, other guidelines were specifically designed to aid in reporting studies incorporating interventions with AI components^33,34^. These frameworks and guidelines primarily concentrated on reporting and regulatory aspects. However, they offered limited guidance on assessing the application of AI systems. A later study proposed Translational Evaluation of Healthcare AI (TEHAI) to guide the evaluation of AI systems integrating in clinical settings and provide a scoring matrix^35^. Yet, TEHAI lacks consideration of pertinent stakeholders and detailed information within each component.

Remarkably, only a few indicators reported in the proposed articles for evaluating AI systems in specialized healthcare clinics are guided by the above existing models or frameworks^20,36^; instead, the indicators were often self-conducted. Some focused on clinician impacts, some on patient impacts, and others on economic impacts, revealing a lack of systematic and comprehensive options^37^. AI-DSS in pediatric healthcare has a more complex application background, encompassing diverse clinical scenarios, treatment possibilities, and a wide age span range from 0 to 18 years. The evaluation process requires to evolve from being a one-time activity to a continuous process, ensuring effective AI-DSS utilization, considering all stakeholders, and providing a comprehensive set of indicators for practical evaluation. This study aims to develop a set of evaluation indicators tailored specifically for AI-DSS in pediatric healthcare, enabling continuous and systematic performance monitoring. It will be established through expert consensus and then integrated into the hospital information system of the Children's Hospital of Fudan University for a pilot implementation. We hypothesize that this incorporated evaluation indicator set will realize dynamic monitoring, and significantly enhance AI governance within pediatric healthcare settings.

## Methods

### Study design

The study was conducted in two stages: (1) Generating the draft of the evaluation indicators via literature reviews and focus group interviews, followed by executing the Delphi study to establish the final indicator set; (2) Conducting weight analysis for the finalized indicators. The study was approved by the Research Ethics Board of Children's Hospital of Fudan University (No. 2022307A), and all methods were carried out in accordance with relevant guidelines and regulations. Informed consent was obtained from all participants.

### Procedures, participants, and data collection

#### Literature review

A systematic literature review of AI decision-making system studies in healthcare was conducted first. We searched Ovid Medline, Ovid Embase, Cochrane Library for eligible English written studies, and China National Knowledge Infrastructure (CNKI), Wanfang data, Sinomed for eligible Chinese written studies published from 1990 to 2022. The following keywords or medical terms were used: artificial intelligence, machine learning, clinical decision support, evaluation, metrics, indicator, index, framework, implementation, application, performance, and success. Considering AI systems deployed in healthcare sectors are generally based on hospital information systems, we also searched literature related to evaluation indicators for information systems. The literature inclusion criteria were: (1) language: English or Chinese; (2) application scenario: healthcare sectors; (3) content: application evaluation for AI systems or hospital information systems. The exclusion criteria included conference abstracts and articles lacking accessible full text.

#### Constructing the draft evaluation indicator set

After the literature review, a focus group interview was conducted to get more information on the evaluation indicators for AI-DSS in healthcare sectors. Eleven participants, actively involved in AI-DSS development research or its utilization, were invited to attend the virtual interview session through the Tengxun APP. They hailed from four tertiary children's hospitals and one university in Shanghai Municipality, Anhui Province, Jiangsu Province, and Shandong Province. Among them were four physicians, two nurses, two radiologists, one pharmacist, and two AI professors. We encouraged them to share their insights on the following topics: (1) What is your interpretation of a successful AI-DSS in pediatric outpatient clinics? (2) What criteria do you consider essential for evaluating AI-DSS in pediatric outpatient clinics?

Eventually, relevant content from literature and interviews was extracted, duplicated and synthesized into indicators by two independent members of the research team. These indicators were subsequently categorized based on the Society-Management-Application-Result-Technology (SMART) model. This model, rooted in recent theoretical and empirical AI implementation studies, was developed through expert consensus within a panel of national AI experts convened by the Shanghai AI Laboratory. The expert panel agreed that evaluating an AI system should encompass three primary dimensions: societal performance (referred to as society), organizational performance (referred to as management), and user experience performance (referred to as application). Within each dimension, various components of technology utility should be assessed (referred to as technology), and the outcomes of these components should be comprehensively analyzed and documented (referred to as result). Significant emphasis was placed on tailoring the evaluation indicator set to suit a specialized application scenario. Further details can be found at https://www.shlab.org.cn/. Consequently, the '1–3-5 evaluation indicator set' was developed for further Delphi study, where '1' signifies the pediatric outpatient clinic, '3' represents the three evaluation dimensions, and '5' denotes the five components of technology utility.

#### Delphi study

Delphi methodology was employed to develop the 1–3–5 evaluation indicator set. Using the purposive sampling method, experts were recruited from members of the Chinese Medical Information and Big Data Association (CHMIA) Pediatric Committee. The CHMIA is a national-level association under the supervision of the National Health Commission of the People’s Republic of China, dedicated to advancing healthcare informatics and big data analytics, fostering collaboration, innovation, and policy advocacy in the fields of health information technology and data science. The Experts inclusion criteria were as follows: (a) having experience in either using or designing AI systems in pediatric healthcare sectors; (b) more than 10 years of working experience; and (c) with high interest, willing to participate in this study.

The Delphi study questionnaire was structured into four sections: (I) introduction of the study and instructions for completing the questionnaire.; (II) demographic information of the expert, including age, gender, training experience, professions, and academic title; (III) draft of the 1–3–5 evaluation indicator set, wherein the experts were asked to rate the importance of each category and indicator, using a 5-point Likert scale, ranging from 'Not at all important (1 point)' to 'Extremely important (5 points)'. Additionally, experts could propose additions, deletions, or modifications; (IV) experts' familiarity with the study and their judgment on the indicators. A total of two rounds of Delphi study were conducted. Experts were asked to complete the questionnaires and returned within 2 weeks. In the first round, paper questionnaires were distributed to the expert panel during the CHMIA annual committee meeting on March 4, 2023. Additionally, mail services were arranged for each expert to facilitate questionnaire returns. All Round 1 questionnaires were promptly received by March 10, 2023, and Round 2 took place from March 13 to March 26, 2023, with all questionnaires distributed and returned via email.

After collecting data from each round, the study team screened out indicators with a mean importance score of ≤ 3.5, a coefficient of variation of ≤ 0.25, and a full mark rate ≤ 20%^38^, while also analyzing experts' opinions. The results were then shared with the experts following each round.

### Determining the index weights

#### Analytic hierarchy process

The rated importance score for each indicator, as provided by each expert, was compiled from the final consultation round. Following this, a judgment matrix was established to conduct pairwise comparisons and importance ratios were then determined using Saaty’s 9-point scale^39^. The weight vector was then measured using yaahp 10.1 software (https://www.metadecsn.com/yaahp/).

#### Entropy weight method

The data sources for collecting the necessary information to calculate the evaluation indicators were identified. Relevant data fields from the hospital information system were mapped, and data extraction mechanisms were developed. Subsequently, data were summarized every quarter, specifically on March 31, 2023, June 30, 2023, and September 28, 2023, at Children’s Hospital of Fudan University, defined as m quarters and n indicators, and the indicators were normalized to obtain the data matrix. ${X}_{ij}$ is the original value of quarter i, indicator j.

As to forward indicators:

$${X}_{ij}=\frac{{X}_{ij}-\text{min}({X}_{ij})}{max\left({X}_{ij}\right)-min({X}_{ij})}$$

As to inverted indicators:

$${X}_{ij}=\frac{{\text{max}\left({X}_{ij}\right)-X}_{ij}}{max\left({X}_{ij}\right)-min({X}_{ij})}$$

The entropy value of indicator j was calculated as follows.

$${\text{En}}_{{\text{j}}} = - {\text{k }} \times \sum\limits_{i = 1}^{m} {\sum\limits_{j = 1}^{n} {P_{ij} } } \, \times {\text{ln}}(P_{ij} ),\,{\text{i}} = 1,2,...,{\text{m}};\,{\text{j}} = 1,2,...,{\text{n}},{\text{k}}\, = \frac{1}{{{\text{ln}}(m \times n)}}$$

The difference coefficient of indicator j was calculated as follows:

$${\text{D}}_{{\text{j}}} = 1 - {\text{En}}_{{\text{j}}} .$$

The weight of indicator j was calculated as follows:

$${\text{W}}_{\text{j}}\hspace{0.17em}=\hspace{0.17em}\frac{\text{Dj}}{\sum_{j=1}^{n}\text{Dj}}$$

#### Combination weight method

Assuming the weight vector calculated by the hierarchical analysis is α = (α_1_, α_2_, ..., α_n_) and the weight vector calculated by the entropy weight method is β = (β_1_, β_2_, ..., β_n_). Combined weights were calculated based on geometric average method, and the calculation formula was as follows^40^:

$${\text{W}}_{\text{j}}=\frac{\sqrt{{\alpha }_{i}{\upbeta }_{j}}}{\sum_{j=1}^{n}\sqrt{{\alpha }_{i}{\upbeta }_{j}}}$$

*The entropy weight and* the combination method were all realized through python, and detailed python code can be found in Supplement A.

### Statistical analysis

SPSS 25.0 statistical software (https://www.ibm.com/support/pages/downloading-ibm-spss-statistics-25), python 3.11 (python 3.11), and Analytic Hierarchy Process (AHP) yaahp 10.1 (https://www.metadecsn.com/yaahp/) were used to analyze the data. The measurement data were described by mean ± standard deviation, and the count data were described for frequency and percentage. The experts' enthusiasm was quantified through the effective recovery rate of the questionnaire, while the expert authority coefficient was determined as the mean of the judgment coefficient and familiarity coefficient. The degree of coordination among expert opinions was assessed using Kendall's harmony coefficient. A minimum recovery rate of 80 percent is indicative of a valid result. An expert authority coefficient exceeding 0.70 suggests high expertise among the experts, lending credibility to the inquiry results. Kendall's coordination coefficient should first be statistically significant (P < 0.05), with values ranging between 0 (indicating no agreement) and 1 (representing complete agreement), where higher values denote stronger agreement, and those below 0.2 suggest poor agreement. The combination of the subjective weighting method (the analytic hierarchy process, AHP) and objective weighting method (the Entropy Weight Method, EWM) were used to calculate the 1–3–5 evaluation indicator set and determine the impact of the assessment indicators using the software Analytic Hierarchy Process (AHP) yaahp 10.1 and python 3.11. The details can be found in the supplement material.

## Results

### Sample characteristics

Fifty-two invitees agreed to participate in the Delphi consultations, with a 100.0% response rate for Round 1 questionnaires and 92.0% for Round 2. Three experts dropped out due to non-response within two weeks. All returned questionnaires were completed. The participating experts come from eight municipalities and provinces in China, including Shanghai, Tianjin, Jiangsu Province, Henan Province, Shandong Province, Hubei Province, Anhui Province, and Zhejiang Province. Demographic characteristics of the experts are shown in Table 1.

**Table 1:** Demographic characteristics of participants in Delphi rounds.

Characteristics	Round 1 (N = 52)	Round 2 (N = 49)
Gender		
Female	23 (44.2%)	21 (42.9%)
Male	29 (55.8%)	28 (57.1%)
Age	41.94 (SD 5.775) years	41.69 (SD 5.587) years
Education		
Bachelor	3 (5.8%)	3 (6.1%)
Master	11 (21.2%)	10 (20.4%)
PhD	38 (73.0%)	36 (73.5%)
Academic title		
Professor/Researcher	21 (40.4%)	21 (42.9%)
Associate Professor/Researcher	31 (59.6%)	28 (57.1%)
Affiliation		
Hospital	36 (69.2%)	34 (69.4%)
University	7 (13.5%)	7 (14.3%)
Technology Company	9 (17.3%)	8 (16.3%)
Occupation Title		
Physician	18 (34.6%)	17 (34.7%)
Pediatric nurse	11 (21.2%)	11 (22.4%)
Radiologist	6 (11.5%)	6 (12.2%)
Pharmacist	3 (5.8%)	3 (6.1%)
AI engineer	8 (15.4%)	7 (14.3%)
AI Algorithm researcher	6 (11.5%)	5 (10.2%)

### Expert authority coefficient and the degree of opinion coordination

In the two rounds of expert consultation, the authority coefficients were 0.834 and 0.846, respectively, which met the criteria of the expert consultation authority coefficient > 0.7. Kendall's coordination coefficient was 0.135 in Round 1 and 0.312 in Round 2. Kendall's test had statistical significance (all p < 0.001).

### 1–3-5 Evaluation indicator set and corresponding weights

The preliminary indicator set included three first-class indicators and fifteen second-class indicators, constructed using the SMART model, along with fifty-six third-class indicators derived from the literature reviews and interview, following deliberation among the research team. In Round 1, no changes were made to the first and second-class indicators. However, five third-class indicators, including ‘coverage rate of disease spectrum’, ‘number of jobs displaced by AI-DSS’, ‘public comprehension of AI-DSS’, ‘daily average patient visits per clinical doctor’, and ‘daily average outpatient and emergency room visits per hospital’, were directly eliminated based on the exclusion criteria. In comparing ‘time expenditure for AI-DSS operation training’ with ‘training frequency for AI-DSS utilization’, the latter was deemed a more reasonable indicator for reflecting the accessibility of user experience performance. Consequently, ‘time expenditure for system operation training’ was eliminated and ‘training frequency for AI-DSS utilization’ was added based on experts’ opinions and research team discussion. Similarly, in comparing ‘number of granted invention patents’, ‘number of granted utility model patents’, ‘number of granted software copyrights’, ‘number of medical device certificates obtained’, ‘quantities of embedded algorithms’ with ‘extent of process reengineering for improved healthcare services via AI-DSS’, the latter was considered more representative of innovation in societal performance. Thus, ‘extent of process reengineering for improved healthcare services via AI-DSS’ was selected to replace the former five indicators following experts’ opinions and research team discussion. Furthermore, seven third-class indicators underwent wording revisions. In Round 2, only three third-class indicators underwent wording revisions, and there were no further deletions from or additions to the indicator set.

Ultimately, 1–3–5 evaluation indicator set including three first-class, fifteen second-class, and forty-seven third-class indicators were established. As depicted in Table 2, seventeen third-class indicators highlighted in bold font are derived from data collected through self-reports of AI-DSS users, leadership, and the general public, using an electric 5-point Likert scale. The remaining third-class indicators are calculated from data directly obtained from hospital information system.

**Table 2:** 1–3–5 Evaluation Indicator set.

First-class Indicator	Second-class Indicator	Third-class Indicator
I-1 User experience performance	II-1 Usefulness	**III-1 Timeliness of the medical information provided by AI-DSS**
		III-2 accuracy of the medical information provided by AI-DSS
		III-3 usability of the medical information provided by AI-DSS
	II-2 Convenience	III-4 Operability of AI-DSS
		III-5 Reliability of AI-DSS
		III-6 Compatibility of AI-DSS with HIS
		III-7 Entire response speed of AI-DSS
	II-3 Accessibility	III-8 Implementation rate of AI-DSS
		III-9 Training frequency for AI-DSS utilization
		III-10 Average resolution time for bug fixes or requests fulfilled during AI-DSS utilization
	II-4 Privacy	III-11 Ratio of alerts generated in AI-DSS data acquisition
		III-12 Accounting for patient individuality, beliefs, and preferences in AI-DSS utilization
	II-5 Satisfaction	**III-13 Satisfaction with AI-DSS functionality**
		**III-14 Satisfaction with AI-DSS flexibility**
		**III-15 Satisfaction with AI-DSS effectiveness**
		**III-16 Satisfaction with AI-DSS**
		**III-17 Satisfaction with AI-DSS safety**
I-2 Organizational performance	II-6 Cost	III-18 Implementation cost of AI-DSS
		III-19 Operational maintenance cost of AI-DSS
		III-20 Training cost for AI-DSS utilization
	II-7 Efficiency	III-21 Average consultation duration for patient in the clinic
		III-22 Average time taken for physician decision-making in the clinic
	II-8 Workability	III-23 alignment ratio of diagnoses between healthcare professionals and AI-DSS
		III-24 False negative diagnosis rate in AI-DSS
		III-25 Ratio of alerts generated for high-risk diseases in AI-DSS
		III-26 Volume of clinical decision support generated by AI-DSS
	II-9 Risk	III-27 Proportion of AI-DSS undergoing ethical review pre-deployment
		**III-28 Algorithm transparency of AI-DSS**
		**III-29 Algorithm interpretability of AI-DSS**
		III-30 Risk level in integrating AI-DSS with hospital information systems
	II-10 Foreground	**III-31 Enhancement of hospital reputation through AI-DSS**
		**III-32 Optimization budget for AI-DSS**
I-3 Societal performance	II-11 Innovation	**III-33 Extent of process reengineering for improved healthcare services via AI-DSS**
		III-34 System update frequency for AI-DSS
	II-12 Effect	III-35 Average patient waiting time
		III-36 Healthcare professionals' compliance with standards and guidelines
		**III-37 Patient satisfaction with healthcare services**
		III-38 Average cost for each outpatient visit
	II-13 Feasibility	**III-39 Extent of relevant policy support at the national level**
		**III-40 Extent of relevant policy support at the local level**
		**III-41 Extent of hospital informatization**
	II-14 Security	III-42 Traceability of data acquisition process
		III-43 Documentation of AI-DSS modifications in Patient Medical Data
		III-44 Incidence Rate of Patient Medical Data Breaches
		III-45 Occurrence of Medical Injuries Linked to AI-DSS
	II-15 Sustainability	**III-46 Trustworthiness of AI-DSS**
		**III-47 Acceptance of AI-DSS**

Indicators derived from data collected through self-reporting.

As detailed in Table 3, all the indicators included in Round 2 exhibited mean scores of importance exceeding 3.5, coefficient of variation surpassing 2.5, and full mark rates exceeding 20%. In terms of weight, societal performance carried the highest weight (0.539), followed by organizational performance (0.164), with user experience performance (0.297) having the smallest weight, according to subjective weighting method. However, under the subjective weighting method, organizational performance held the highest weight (0.690), societal performance followed by (0.302), with user experience performance (0.008) having the smallest weight. Similarly, in the combined weighting method, organizational performance retained the highest weight (0.543), societal performance followed (0.391), and user experience performance (0.067) had the smallest weight, following. After rounding to three decimal places, many values of 0.000 emerged in the objective weights column, indicating minimal variation observed in both the data collected from self-reports of AI-DSS users and the data derived directly from hospital information system each quarter.

**Table 3:** Weights for indicators.

Indicators	Importance of round 2				AHP weights	Entropy weights	Combined weights
	Mean	SD	CV	Full mark rate			
**I-1**	4.755	0.516	0.109	79.6	0.164	0.008	0.067
II-1	4.837	0.370	0.076	83.7	0.020	0.000	0.007
III-1	4.469	0.703	0.157	57.7	0.011	0.000	0.004
III-2	4.755	0.554	0.117	79.6	0.003	0.000	0.001
III-3	4.653	0.517	0.111	67.3	0.006	0.000	0.002
II-2	4.837	0.421	0.087	85.7	0.020	0.000	0.009
III-4	4.837	0.370	0.076	83.7	0.004	0.000	0.002
III-5	4.857	0.404	0.083	87.8	0.003	0.000	0.002
III-6	4.612	0.565	0.122	65.3	0.008	0.000	0.003
III-7	4.633	0.560	0.121	67.3	0.006	0.000	0.002
II-3	4.776	0.417	0.087	77.6	0.031	0.002	0.013
III-8	4.429	0.639	0.144	51.0	0.008	0.000	0.003
III-9	4.102	0.735	0.179	30.6	0.019	0.002	0.008
III-10	4.612	0.600	0.130	67.3	0.004	0.000	0.002
II-4	4.612	0.600	0.130	67.3	0.046	0.000	0.018
III-11	4.612	0.633	0.137	69.4	0.015	0.000	0.006
III-12	4.592	0.636	0.139	67.3	0.031	0.000	0.012
II-5	4.612	0.565	0.122	65.3	0.046	0.006	0.020
III-13	4.612	0.527	0.114	63.3	0.013	0.001	0.005
III-14	4.714	0.452	0.096	71.4	0.007	0.001	0.003
III-15	4.694	0.503	0.107	71.4	0.005	0.001	0.002
III-16	4.633	0.629	0.136	69.4	0.009	0.002	0.005
III-17	4.612	0.600	0.130	67.3	0.013	0.001	0.005
**I-2**	4.551	0.608	0.134	61.2	0.297	0.690	0.543
II-6	4.327	0.739	0.171	49	0.105	0.397	0.286
III-18	4.449	0.672	0.151	55.1	0.026	0.120	0.083
III-19	4.449	0.672	0.151	55.1	0.026	0.000	0.010
III-20	4.265	0.722	0.169	42.9	0.052	0.277	0.193
II-7	4.755	0.516	0.109	79.6	0.034	0.287	0.193
III-21	4.510	0.674	0.149	61.2	0.017	0.159	0.106
III-22	4.510	0.643	0.143	59.2	0.017	0.128	0.087
II-8	4.796	0.451	0.094	81.6	0.024	0.006	0.013
III-23	4.755	0.475	0.100	77.6	0.003	0.000	0.001
III-24	4.694	0.579	0.123	75.5	0.007	0.000	0.003
III-25	4.735	0.486	0.103	75.5	0.005	0.000	0.002
III-26	4.571	0.639	0.140	65.3	0.009	0.006	0.007
II-9	4.531	0.610	0.135	59.2	0.056	0.000	0.021
III-27	4.592	0.569	0.124	63.3	0.009	0.000	0.003
III-28	4.367	0.774	0.177	53.1	0.016	0.000	0.006
III-29	4.265	0.875	0.205	51	0.025	0.000	0.010
III-30	4.633	0.691	0.149	73.5	0.006	0.000	0.002
II-10	4.490	0.610	0.136	55.1	0.079	0.000	0.030
III-31	4.265	0.777	0.182	38.8	0.053	0.000	0.020
III-32	4.347	0.846	0.195	51	0.027	0.000	0.010
**I-3**	4.469	0.835	0.187	63.3	0.539	0.302	0.391
II-11	4.367	0.874	0.200	55.1	0.206	0.000	0.076
III-33	4.327	0.866	0.200	53.1	0.154	0.000	0.057
III-34	4.245	0.770	0.181	42.9	0.052	0.000	0.019
II-12	4.694	0.706	0.150	77.6	0.052	0.289	0.200
III-35	4.653	0.716	0.154	73.5	0.007	0.141	0.091
III-36	4.571	0.756	0.165	67.3	0.014	0.000	0.005
III-37	4.612	0.723	0.157	69.4	0.010	0.000	0.004
III-38	4.490	0.811	0.181	63.3	0.020	0.148	0.100
II-13	4.653	0.744	0.160	75.5	0.086	0.000	0.033
III-39	4.592	0.754	0.164	69.4	0.017	0.000	0.007
III-40	4.551	0.758	0.166	65.3	0.027	0.000	0.010
III-41	4.469	0.759	0.170	57.1	0.042	0.000	0.016
II-14	4.694	0.706	0.150	77.6	0.052	0.013	0.028
III-42	4.673	0.585	0.125	73.5	0.017	0.000	0.006
III-43	4.673	0.585	0.125	73.5	0.017	0.013	0.015
III-44	4.755	0.475	0.100	77.6	0.011	0.000	0.004
III-45	4.776	0.506	0.106	81.6	0.007	0.0000	0.003
II-15	4.429	0.808	0.182	57.1	0.143	0.000	0.054
III-46	4.612	0.565	0.122	65.3	0.048	0.000	0.018
III-47	4.510	0.674	0.149	61.2	0.095	0.000	0.036

## Discussion

The number of publications describing the applications of AI systems in healthcare settings has snowballed over the past decade; the majority solely report on their effects^41^, and the actual impact when deploying AI in clinical settings remains largely unknown^42^. At the same time, evidence supporting clinical effectiveness, comparative effectiveness, cost-effectiveness, or other formal health technology assessment of AI in a clinical healthcare setting appears to be limited^43^. A recent systematic review highlighted a key reason for the lack of adoption of AI systems: the absence of demonstrated benefits. The risk lies in adopting AI systems with insufficient evidence, potentially leading to the incorporation of non-valuable or inadequately supported systems^44^.

In response to these challenges, researchers have developed multiple models and frameworks aimed at elucidating the elements contributing to AI systems. These model and framework-guided tools played an essential role in evaluating the AI systems^45^. Indeed, these models have limitations due to their specific focus. Some center on assessing AI algorithms in controlled laboratory settings^46^, while others aim to offer holistic metrics for evaluating user experience in clinical settings^36,37^. AI systems evaluation should not only start early in the development process but also be continuous and comprehensive, considering the ethical implications introduced by AI. This comprehensive approach is often lacking in current evaluation frameworks.

In this study, a comprehensive set of evaluation indicators, specifically tailored for AI-DSS implementation in pediatric outpatient clinics, was developed using the Delphi method. The draft was based on the established model specific to AI system performance evaluation and information extracted from the relevant published studies. The 49 consulting experts came from 8 provinces and cities across China, and they were all experienced in the field of implementing AI systems in pediatrics. The set of evaluation indicators has a strong foundation in the pediatric outpatient clinic and emphasizes the measurable results for the technology application in three dimensions. Compared with the TEHAI^35^, this set is tailored explicitly for pediatric outpatient clinics and offers a more extensive range of detailed indicators to interpret the success of implementing AI systems. Other reported indicators for evaluating the specialized healthcare clinic were often self-conducted and did not align with models or frameworks^8,9,20,37^.

Regarding weight analysis, previous studies have primarily relied on experts' opinions (subjective weights), utilizing the AHP method^47,48^. However, this study employed real-world data directly captured from the hospital information system to derive objective weights through the entropy weight method. Subsequently, subject and object weights were synthesized to form the combined weight. Incorporating the evaluation indicator set into the hospital information system helps realize active surveillance of the effect of AI-DSS in the pediatric outpatient clinic. The study revealed that the combined weights align much more closely with the objective weights. Indicator I-1(Organizational performance) carries the highest weight, followed by Indicator I-2(Societal performance) and Indicator I-3(User experience performance) in the objective and combined weights. Conversely, among the subjective weights, 'Societal performance' holds the most weight, followed by 'Organizational performance' and 'User experience performance'. The reasons for this discrepancy may stem from the entropy weight method utilized to calculate objective weights. According to its algorithm, if the collected data undergoes only slight changes during observation, the indicator may exhibit an extremely low weight. Indicators III-1 ~ III-4, III-6 ~ III-8, III-6 ~ III-8, III-19, III-23 ~ III-34, III-36 ~ III-37, III-39 ~ III-42, III-44 ~ III-47 exhibited this behavior. Their change trend was curved, offering minimal contribution to reflecting the dynamic performance of the AI system, suggesting a need for prolonged observation or frequent data collection.

### Limitations of the study

Firstly, the Delphi method, being a structured and iterative forecasting technique, depends on the input of a panel of experts. The second round of consulting conducted via email lacks real-time discussion, potentially limiting the depth of analysis and the exploration of alternative viewpoints. Secondly, the subjective weight analysis heavily relies on the expertise and judgment of the participants. Experts' inherent biases, insufficient information, or influenced professional interests can introduce errors or inaccuracies into the forecasting process, potentially affecting the validity of the results. Thirdly, the entropy weight method requires long-term observation or more frequent data collection. The data collected quarterly, and four times may result in bias. Additionally, further exploration of the entropy weight method could involve assessing the impact of different data collection frequencies on the validity of results. Conducting comparative studies with varied observation periods, such as monthly or biannually, could provide insights into the optimal data collection frequency for minimizing bias and enhancing the robustness of the analysis.

## Conclusion

Monitoring and evaluating AI systems in pediatric outpatient clinics necessitates a fitting set of evaluation indicators. The 1–3–5 evaluation indicator set, owing to its comprehensive nature and successful integration into hospital information systems, enables the automatic acquisition of real-world data. This allows for the continuous evaluation and monitoring of AI system performance in pediatric outpatient clinics. Future efforts should focus on enhancing long-term data collection for the indicators to optimize their weight proportions.

### Supplementary Information

Supplementary Information.

## Data Availability

Correspondence and requests for materials should be addressed to X.B.Z.

## References

[1] Administration., U. F. a. D. FDA permits marketing of artificial intelligence-based device to detect certain diabetes-related eye problems: FDA news release. https://www.fda.gov/newsevents/newsroom/pressannouncements/ucm604357.htm. Published April 11, 2018. Accessed 17 April 2024.

[2] He J (2019). The practical implementation of artificial intelligence technologies in medicine. Nat. Med..

[3] Sugano K, Moss SF, Kuipers EJ (2023). Gastric intestinal metaplasia: Real culprit or innocent bystander as a precancerous condition for gastric cancer?. Gastroenterology.

[4] Vishwanathaiah S, Fageeh HN, Khanagar SB, Maganur PC (2023). Artificial intelligence its uses and application in pediatric dentistry: A review. Biomedicines.

[5] Liang H (2019). Evaluation and accurate diagnoses of pediatric diseases using artificial intelligence. Nat. Med..

[6] Dillman JR, Somasundaram E, Brady SL, He L (2022). Current and emerging artificial intelligence applications for pediatric abdominal imaging. Pediatr. Radiol..

[7] Balkenende L, Teuwen J, Mann RM (2022). Application of deep learning in breast cancer imaging. Semin. Nucl. Med..

[8] Radici L (2022). Implementation of a commercial deep learning-based auto segmentation software in radiotherapy: Evaluation of effectiveness and impact on workflow. Life (Basel).

[9] Seibert K (2021). Application scenarios for artificial intelligence in nursing care: Rapid review. J. Med. Internet Res..

[10] Gu Y (2023). Effective multidimensional approach for practical management of the emergency department in a COVID-19 designated children's hospital in east China during the Omicron pandemic: A cross-sectional study. Transl. Pediatr..

[11] Li WH (2023). Artificial intelligence promotes shared decision-making through recommending tests to febrile pediatric outpatients. World J. Emerg. Med..

[12] Li X (2021). Artificial intelligence-assisted reduction in patients' waiting time for outpatient process: A retrospective cohort study. BMC Health Serv. Res..

[13] Nsoesie EO (2018). Evaluating artificial intelligence applications in clinical settings. JAMA Netw. Open.

[14] Yu VL (1979). Evaluating the performance of a computer-based consultant. Comput. Programs Biomed..

[15] Wyatt J, Spiegelhalter D (1990). Evaluating medical expert systems: What to test and how?. Med. Inform. (Lond.).

[16] Nykänen P, Chowdhury S, Wigertz O (1991). Evaluation of decision support systems in medicine. Comput. Methods Programs Biomed..

[17] Clarke K (1994). A methodology for evaluation of knowledge-based systems in medicine. Artif. Intell. Med..

[18] van Gennip EM, Talmon JL, Bakker AR (1994). ATIM, accompanying measure on the assessment of information technology in medicine. Comput. Methods Programs Biomed..

[19] Kanagasingam Y (2018). Evaluation of artificial intelligence-based grading of diabetic retinopathy in primary care. JAMA Netw. Open.

[20] Ciecierski-Holmes T, Singh R, Axt M, Brenner S, Barteit S (2022). Artificial intelligence for strengthening healthcare systems in low- and middle-income countries: a systematic scoping review. NPJ Digit. Med..

[21] Scott I, Carter S, Coiera E (2021). Clinician checklist for assessing suitability of machine learning applications in healthcare. BMJ Health Care Inform..

[22] Cabitza F, Campagner A (2021). The need to separate the wheat from the chaff in medical informatics: Introducing a comprehensive checklist for the (self)-assessment of medical AI studies. Int. J. Med. Inform..

[23] DeLone WH, McLean ER (2003). The DeLone and McLean model of information systems success: A ten-year update. J. Manag. Inform. Syst..

[24] DeLone WH, McLean ER (1992). Information systems success: The quest for the dependent variable. Inf. Syst. Res..

[25] Nguyen L, Bellucci E, Nguyen LT (2014). Electronic health records implementation: An evaluation of information system impact and contingency factors. Int. J. Med. Inform..

[26] Shim M, Jo HS (2020). What quality factors matter in enhancing the perceived benefits of online health information sites? Application of the updated DeLone and McLean information systems success model. Int. J. Med. Inform..

[27] Bossen C, Jensen LG, Udsen FW (2013). Evaluation of a comprehensive EHR based on the DeLone and McLean model for IS success: Approach, results, and success factors. Int. J. Med. Inform..

[28] Cho KW (2015). Performance evaluation of public hospital information systems by the information system success model. Healthc. Inform. Res..

[29] Yang MH (2022). A comparison of two cross-sectional studies on successful model of introducing nursing information system in a regional teaching hospital in Taiwan. Comput. Inform. Nurs..

[30] Salahuddin L, Ismail Z (2015). Classification of antecedents towards safety use of health information technology: A systematic review. Int. J. Med. Inform..

[31] Tubaishat A (2017). Evaluation of electronic health record implementation in hospitals. Comput. Inform. Nurs..

[32] Nykänen P (2011). Guideline for good evaluation practice in health informatics (GEP-HI). Int. J. Med. Inform..

[33] Liu X, Cruz Rivera S, Moher D, Calvert MJ, Denniston AK (2020). Reporting guidelines for clinical trial reports for interventions involving artificial intelligence: The CONSORT-AI extension. Lancet Digit. Health.

[34] Rivera SC, Liu X, Chan AW, Denniston AK, Calvert MJ (2020). Guidelines for clinical trial protocols for interventions involving artificial intelligence: The SPIRIT-AI extension. Bmj.

[35] Reddy S (2021). Evaluation framework to guide implementation of AI systems into healthcare settings. BMJ Health Care Inform..

[36] Parasa S (2023). Framework and metrics for the clinical use and implementation of artificial intelligence algorithms into endoscopy practice: Recommendations from the American Society for Gastrointestinal Endoscopy Artificial Intelligence Task Force. Gastrointest. Endosc..

[37] Yin J, Ngiam KY, Teo HH (2021). Role of artificial intelligence applications in real-life clinical practice: Systematic review. J. Med. Internet Res..

[38] Diamond IR (2014). Defining consensus: A systematic review recommends methodologic criteria for reporting of Delphi studies. J. Clin. Epidemiol..

[39] Saaty TL, Bennett JP (1977). A theory of analytical hierarchies applied to political candidacy. Behav. Sci..

[40] Le-hong, K., Lin-rong, X. & Bao-chen, L. *2006 International Conference on Computational Intelligence and Security.* 963–967.

[41] Gore JC (2020). Artificial intelligence in medical imaging. Magn. Reson. Imaging.

[42] Zhou Q, Chen ZH, Cao YH, Peng S (2021). Clinical impact and quality of randomized controlled trials involving interventions evaluating artificial intelligence prediction tools: a systematic review. NPJ Digit. Med..

[43] Wiegand T, L. N., Pujari S, et al. White paper for the ITU/WHO Focus Group on artificial intelligence for health. ITU. https://www.itu.int/go/fgai4h. Accessed 24 May 2021.

[44] Voets MM, Veltman J, Slump CH, Siesling S, Koffijberg H (2022). Systematic review of health economic evaluations focused on artificial intelligence in healthcare: The tortoise and the cheetah. Value Health.

[45] Urbach N, Smolnik S, Riempp G (2009). The state of research on information systems success. Bus. Inf. Syst. Eng..

[46] Larson DB (2021). Regulatory frameworks for development and evaluation of artificial intelligence-based diagnostic imaging algorithms: Summary and recommendations. J. Am. Coll. Radiol..

[47] Ni X (2021). Development of an evaluation indicator system for the rational use of proton pump inhibitors in pediatric intensive care units: An application of Delphi method. Medicine (Baltimore).

[48] Wei J (2022). Construction on teaching quality evaluation indicator system of multi-disciplinary team (MDT) clinical nursing practice in China: A Delphi study. Nurse Educ. Pract..

