# Supplementary material for: Constructing and implementing a performance evaluation indicator set for artificial intelligence decision support systems in pediatric outpatient clinics: an observational study
Source: Sci Rep. 2024 Jun 24;14:14482. doi: 10.1038/s41598-024-64893-w (PMC11196575; doi:10.1038/s41598-024-64893-w)
Supplement: Supplementary file 1 — Supplementary Information. [file 41598_2024_64893_MOESM1_ESM.pdf]

Python code

```
def Entory(path0, forwrd_indicator, inverse_indicator):
        df2 = pd.read_excel(path0, sheet_name="Sheet1", index_col=0)
        print(df2)

        df4 = df2.copy()
        print(df4)
        # Sheet_name = ['']
        forwrd_indicator = [i for i in forwrd_indicator]
        print("正向指标 forwrd_indicator：\n", forwrd_indicator)
        print("\n")
        print("逆向指标 inverse_indicator：\n", inverse_indicator)
        print("\n")

        print(type(forwrd_indicator))
        print(df4[forwrd_indicator])
        print(type(df4[forwrd_indicator]))
        print(df4[inverse_indicator])
        print(df4[inverse_indicator].max())
        print(df4[inverse_indicator].max() - df4[inverse_indicator])
        print(df4[inverse_indicator].max()-df4[inverse_indicator].min())


        if forwrd_indicator or inverse_indicator:
                df4[forwrd_indicator] = (df4[forwrd_indicator]) / 5
                df4[inverse_indicator] = (df4[inverse_indicator].max() - df4[inverse_indicator]) /
(df4[inverse_indicator].max() - df4[inverse_indicator].min())

        df4 = df4.apply(lambda x: x + 0.01)
        df5 = df4 / df4.apply(lambda x: x.sum())
        k = np.power(np.log(df5.shape[0]), -1)
        p = df5 / df5.apply(lambda x: x.sum())
        P = (p * p.apply(np.log)).sum()
        entory = -k * P    # 计算各列的熵值
        D = 1 - entory    # 计算各指标熵值的差异系数
        W = D / D.sum()    # 计算各指标权重
        print("权重：\n", W)
        print("\n")

        # 将数据和结果写入 Excel 表格
        excel_to_path = os.path.join(os.path.split(path0)[0], "熵值法 111.xlsx")
        with pd.ExcelWriter(path=excel_to_path) as writer:
                # df.to_excel(writer, sheet_name='面板数据')
```

```
        W.to_excel(writer, sheet_name='权重')
    print("结果已保存到路径{}下".format(excel_to_path))


if __name__ == '__main__':
    path0 = r"C:\Users\josh-\Desktop\index.xlsx"   #  目标文件路径(自己填)
    excel = pd.read_excel(path0, sheet_name="Sheet1", index_col=0)
    excel_list = excel.columns.tolist()
    forwrd_indicator = [] #  所有正向指标。
    inverse_indicator = ["系统建设成本", "使用者培训成本", "每位患者平均接诊时间", "每位
患者平均诊断时间", "患者平均候诊时间", "疾病的均次诊疗费用"]   #  负向指标
    for x in excel_list:
        if x not in inverse_indicator and x != "系统维护成本" and x != "响应速度":
        # if x not in inverse_indicator :
            forwrd_indicator.append(x)
    print(forwrd_indicator)
    Entory(path0, forwrd_indicator, inverse_indicator)

    ef Entory(path0):
        df = pd.DataFrame()   #  创建空的 DataFrame
        df1 = pd.DataFrame()   #  创建空的 DataFrame

        sigma1, sigma2 = symbols('sigma1 sigma2')

        df2 = pd.read_excel(path0, sheet_name='权重', index_col=0)   #  读取原始数据，即逐
个读取 sheet
        print(df2)
        w1 = df2.iloc[:, 0]
        w2 = df2.iloc[:, 1]
        w1 = w1.to_numpy()
        w2 = w2.to_numpy()
        w1w1t = np.dot(w1, w1.T)
        w2w2t = np.dot(w2, w2.T)
        w1w2t = np.dot(w1, w2.T)
        w2w1t = np.dot(w2, w1.T)

        eq1 = Eq(w1w1t*sigma1+w1w2t*sigma2, w1w1t)
        eq2 = Eq(w2w1t*sigma1+w2w2t*sigma2, w2w2t)

        solution = solve((eq1, eq2), (sigma1, sigma2))

        sigma1_ = solution[sigma1]/(solution[sigma1]+solution[sigma2])
        sigma2_ = solution[sigma2]/(solution[sigma1]+solution[sigma2])
        wt = sigma1_* w1 +sigma2_ *w2
```

```
    print(wt)

if __name__ == '__main__':
    path0=r"C:\Users\josh-\Desktop\熵值法111.xlsx"#目标文件路径(自己填)
    Entory(path0)
```
